# Has the Number of Pediatric Testicular Torsion Cases Abated After the Height of the COVID-19 Pandemic?

**DOI:** 10.7759/cureus.101735

**Published:** 2026-01-17

**Authors:** Lisa B Shields, Kahir Jawad, Eran Rosenberg

**Affiliations:** 1 Norton Neuroscience Institute, Norton Healthcare, Louisville, USA; 2 Norton Research Institute, Norton Healthcare, Louisville, USA; 3 Pediatric Urology, Norton Healthcare, Louisville, USA

**Keywords:** covid-19, orchiectomy, orchiopexy, pediatric surgery, pediatric urology, testicular torsion

## Abstract

Background: Starting in March 2020, the COVID-19 pandemic represented an international public health emergency which greatly impacted healthcare delivery.

Materials and methods: This retrospective analysis of pediatric testicular torsion (TT) over a 10-year period (January 1, 2015- December 31, 2024) was performed at a pediatric children's hospital in a metropolitan community. We divided our cohort into patients who were evaluated prior to COVID-19 (January 1, 2015-February 29, 2020), during COVID-19 (March 1, 2020-April 30, 2023), and after COVID-19 (March 1, 2023-December 31, 2024). We hypothesized whether the TT incidence and orchiectomy rates differed across these three time periods.

Results: A total of 286 pediatric patients underwent surgery for TT: 101 (35%) before the start of COVID-19, 129 (45%) during COVID-19, and 56 (20%) after the peak of the COVID-19 pandemic. The mean monthly case volume significantly increased from the pre-COVID-19 period (2.06) to during-COVID-19 (3.69) and post-COVID-19 (2.95) periods (p<0.0001). The median door-to-detorsion time (DTD) was significantly different across these periods, with the shortest DTD occurring during the COVID-19 period (p=0.011). The type of surgery (orchiectomy vs. orchiopexy) and symptom duration were not statistically different between the three time periods.

Conclusions: The number of patients with TT significantly increased during the height of the COVID-19 pandemic, with a subsequent reduction. Our study indicates that pediatric patients and their families realized the importance of seeking prompt medical attention for TT despite the ongoing havoc incurred by the COVID-19 pandemic.

## Introduction

Initially affecting patients in Wuhan, China, on December 12, 2019, the first case of the severe acute respiratory syndrome coronavirus 2 (SARS-CoV-2) virus in the United States was reported on January 20, 2020, in the state of Washington [1]. On March 11, 2020, the World Health Organization (WHO) declared the COVID-19 outbreak a pandemic. By February 2023, 755 million COVID-19 cases and 6.8 million deaths had been reported [2]. On May 5, 2023, the WHO declared an end to the public health emergency of international concern. While COVID-19 has not been eradicated, the number of cases and fatalities has subsequently decreased significantly.

During the COVID-19 pandemic, many pediatric units in hospitals were repurposed to expand adult capacity or shuttered which impacted access to pediatric hospital care [3-5]. Pediatric urological emergencies such as testicular torsion (TT) require rapid evaluation in the emergency department (ED) to reduce the risk of an ischemic testis and the need for an orchiectomy. The critical window for TT is between four and six hours from symptom initiation to surgery, after which the possibility of undergoing an orchiectomy is greatly increased [6,7]. The likelihood of testicular salvage reduces to less than 10% if the TT has persisted for more than 24 hours [6]. Due to the urgent nature of this condition, the availability of pediatric hospitals staffed with pediatric urologists or pediatric surgeons who are well-equipped to perform this surgery in a timely manner is imperative, even in the face of a global pandemic. Few studies have investigated the impact of the COVID-19 pandemic on the number of pediatric TT cases and orchiectomies performed as well as the duration of symptoms [8-18]. These studies shared their own experiences with pediatric TT and represented a host of countries worldwide, including the United States [12,14,16-18], Canada [16], the United Kingdom [15], Ireland [11], Italy [9], Poland [13], Croatia [10], and China [8].

We previously described the surge in TT cases in the early months of the COVID-19 pandemic compared to the same months in the five previous years [14]. In addition to more cases of TT during the COVID-19 pandemic, patients also had a longer duration of symptoms and underwent a higher number of orchiectomies. We have expanded upon our previous study by greatly increasing the number of patients with TT and assessing pediatric TT throughout the duration of the COVID-19 pandemic and after the conclusion of the public health emergency. Herein, we compare the number of patients with TT and the type of surgery performed before, during, and after the peak of the COVID-19 pandemic. The number of patients with TT evaluated at our pediatric acute care children's hospital in relation to the fluctuations in the reported COVID-19 cases in our state of Kentucky is highlighted.

The primary goal of this study was to compare the monthly TT case rates before, during, and after the COVID-19 pandemic. The secondary goals of the study were to assess door-to-detorsion time (DTD) and orchiectomy rates before, during, and after the COVID-19 pandemic. We hypothesized whether the TT incidence and orchiectomy rates differed across these three time periods.

## Materials and methods

Under an IRB-exempt protocol and conforming to the Declaration of Helsinki, we identified male children and adolescents aged 1-20 years who underwent surgery for TT over a 10-year period (January 1, 2015-December 31, 2024) at Norton Children's Hospital in Louisville, Kentucky. The ICD10 code used to search for TT surgery was N44.00. The electronic health record system EPIC at our institution was utilized to obtain the patients' data. Our cohort was divided into patients who had surgery for TT prior to (January 1, 2015-February 29, 2020), during (March 1, 2020-April 30, 2023), and after COVID-19 (May 1, 2023-December 31, 2024). The date of April 30, 2023, was determined to be the cutoff date for the end of the COVID-19 period since the WHO emergency ended five days later. We wanted to use the official WHO emergency date as the end of the COVID-19 period since this applied to all individuals worldwide. We also compared two groups: patients in the pre-COVID-19 period (January 1, 2015-February 29, 2020) to patients during and after the peak of COVID-19 (March 1, 2020-December 31, 2024).

The inclusion criteria included male children and adolescents who were aged 1-20 years and underwent surgery for TT. All patients evaluated during the 10-year period (January 1, 2015-December 31, 2024) and who fit the inclusion criteria were included in this study. The exclusion criteria included patients with neonatal TT (TT that occurs in utero up to the first 30 days after birth) as well as patients less than one year of age or older than 20 years of age. Patients were also excluded if they exhibited signs of acute scrotum without evidence of TT. Therefore, we did not record data on patients who presented with testicular pain who were not diagnosed with TT.

A pediatric urologist obtained the medical history and performed a genitourinary physical examination in our institution's ED. All patients underwent a Doppler scrotal ultrasound (US). TT was confirmed based on the lack of blood flow to the affected testis by scrotal US. Several characteristics were obtained, including the patients' age, duration and side of symptoms, type of surgery (orchiectomy versus orchiopexy), DTD (duration between the start of ED triage and initiation of surgical anesthesia), and degree of TT noted intraoperatively. The degree of TT observed during surgery followed the standardized torsion degree measurement protocol.

Statistical analysis

Descriptive statistics summarized the cohort characteristics. Continuous variables, assessed for normality using the Shapiro-Wilk test, were reported as median (Q1, Q3), while categorical variables were presented as frequencies and percentages. The distributions of continuous variables were assessed using Shapiro-Wilk tests and visual inspection of histograms. All time-based variables (symptom duration, DTD) were right-skewed; therefore, medians and interquartile ranges are reported, and non-parametric tests (Wilcoxon rank-sum) were used for bivariate comparisons. Chi-squared tests were used for categorical variables, the Cochran-Armitage test was used for ordinal variables, and the Wilcoxon rank-sum test or Kruskal-Wallis test, t-test, and analysis of variance (ANOVA) were used for continuous variables. Multivariable logistic regression models examined the association of the COVID-19 period with the type of surgery, controlling for demographic and clinical covariates. Variables for multivariable analysis were selected using a combined approach. The COVID-19 period (pre vs. post) was included in all models as the primary exposure of interest for this study, irrespective of its univariate significance. Additional covariates were selected based on clinical relevance and a univariate screening threshold of p<0.20. A hierarchical modeling approach was used, with backward elimination (retention threshold p<0.10) while forcing in the COVID-19 period variable. Variables were tested for effect modification using interaction terms. Multicollinearity was assessed using variance inflation factors (VIF); all were below 1.1, indicating no concerning collinearity among predictors. All analyses were performed using SAS Version 9.4 (SAS Inc., Cary, North Carolina, United States), with statistical significance set at p<0.05.

Institutional review board

The University of Louisville Institutional Review Board determined that this study was exempt according to 45 CFR 46.101(b) under Category 4. The IRB number is 20.0778, and the approval date was September 16, 2020.

## Results

Three time periods of the COVID-19 pandemic (three groups)

A total of 286 pediatric patients underwent surgery for TT: 101 (35%) before the start of COVID-19, 129 (45%) during COVID-19, and 56 (20%) after the peak of the COVID-19 pandemic. The mean monthly case volume was significantly different across the three periods, with the average monthly case volume significantly increasing from the pre-COVID-19 period (2.06) to during-COVID-19 (3.69) and post-COVID-19 (2.95) periods (p<0.0001) (Table 1). 

**Table 1:** Patient characteristics with testicular torsion before, during, and after the peak of COVID-19 divided by three groups Data presented as median (interquartile range), mean±standard deviation, or n (%). ^a^H=Kruskal-Wallis test statistic (df=2); ^b^χ²=Pearson's chi-squared test statistic (df=2); ^c^M²=Cochran-Mantel-Haenszel test statistic (df=1); ^d^F=ANOVA test statistic (df=2). Bolded p-values indicate statistical significance (p<0.05).

	Total	Pre-COVID-19 (1/1/15-2/29/20)	During COVID-19 (3/1/20-4/30/23)	Post-COVID-19 (5/1/23-12/31/24)	Test statistics	P-value
Overall	N=286	101 (35%)	129 (45%)	56 (20%)		
Average number of cases per month	2.78±1.62	2.06±0.90	3.69±1.98	2.95±1.51	F(2)=12.79^d^	<0.0001^d^
Age (yr)	14 (13, 16)	14 (13, 16)	14 (13, 15)	15 (13, 17)	H(2)=5.58^a^	0.060^a^
Duration of symptoms (hr)	9 (4, 48)	9 (5, 48)	8 (4, 36)	13 (5, 48)	H(2)=3.12^a^	0.210^a^
Duration of symptoms						
0 to <6 hours	99 (34.6%)	31 (30.7%)	53 (41.1%)	15 (26.8%)	M²(1)=0.85^c^	0.357^c^
6 to <12 hours	54 (18.9%)	27 (26.7%)	18 (14%)	9 (16.1%)		
12 to <18 hours	27 (9.4%)	8 (7.9%)	13 (10.1%)	6 (10.7%)		
18 to <24 hours	13 (4.6%)	4 (4%)	5 (3.9%)	4 (7.1%)		
24 to <48 hours	20 (7%)	4 (4%)	9 (7%)	7 (12.5%)		
≥48 hours	73 (25.5%)	27 (26.7%)	31 (24%)	15 (26.8%)		
Duration of symptoms						
<24 hours	193 (67.5%)	70 (69.3%)	89 (69%)	34 (60.7%)	χ²(2)=1.46^b^	0.483^b^
≥24 hours	93 (32.5%)	31 (30.7%)	40 (31%)	22 (39.3%)		
Door-to-detorsion time (mins)	147 (97, 193)	165 (117, 208)	132 (86, 166)	157 (88, 195)	H(2)=9.07^a^	0.011^a^
Degree of torsion						
0°	36 (15.3%)	10 (11.6%)	19 (19%)	7 (14%)	M²(1)=0.97^c^	0.326^c^
180°	32 (13.6%)	10 (11.6%)	13 (13%)	9 (18%)		
300-450°	99 (41.9%)	39 (45.4%)	40 (40%)	20 (40%)		
>540°	69 (29.2%)	27 (31.4%)	28 (28%)	14 (28%)		
Side						
Left	135 (47.2%)	48 (47.5%)	52 (40.3%)	35 (62.5%)	χ²(2)=7.72^b^	0.021^b^
Right	151 (52.8%)	53 (52.5%)	77 (59.7%)	21 (37.5%)		
Type of surgery						
Orchiopexy	195 (68.2%)	64 (63.4%)	91 (70.5%)	40 (71.4%)	χ²(2)=1.68^b^	0.431^b^
Orchiectomy	91 (31.8%)	37 (36.6%)	38 (29.5%)	16 (28.6%)		

The median DTD was significantly different across the three periods, with the shortest time occurring during the COVID-19 period (p=0.011). 

Characteristics of patients with TT comparing pre- and during/post-COVID-19: univariate analysis of two groups

The mean number of patients who underwent surgery for TT per month before COVID-19 (2.06±0.90) significantly increased during/after the peak of COVID-19 (3.43±1.85) (p<0.0001) (Table 2). 

**Table 2:** Patient characteristics with testicular torsion before and during/after the peak of COVID-19 divided by two groups Data presented as median (interquartile range), mean±standard deviation, or n (%). ^a^Z=Wilcoxon rank-sum test statistic; ^b^χ²=Pearson's chi-squared test statistic (df=1); ^c^Z=Cochran-Armitage trend test statistic; ^d^t=two-sample t-test statistic (df=101). Bolded p-values indicate statistical significance (p<0.05).

	Total	Pre-COVID-19 (1/1/15-2/29/20)	Post-COVID-19 (3/1/20-12/31/24)	Test statistics	P-value
Overall	N=286	101 (35%)	185 (65%)		
Average number of cases per month	2.78±1.62	2.06±0.90	3.43±1.85	t(101)=-4.69^d^	<0.0001
Age (yr)	14 (13, 16)	14 (13, 16)	14 (13, 16)	Z=-0.72^a^	0.474
Duration of symptoms (hr)	9 (4, 48)	9 (5, 48)	9 (4, 41)	Z=0.69^a^	0.489
Duration of symptoms					
0 to <6 hours	99 (34.6%)	31 (30.7%)	68 (36.8%)	Z=-0.19^c^	0.134
6 to <12 hours	54 (18.9%)	27 (26.7%)	27 (14.6%)		
12 to <18 hours	27 (9.4%)	8 (7.9%)	19 (10.3%)		
18 to <24 hours	13 (4.6%)	4 (4%)	9 (4.9%)		
24 to <48 hours	20 (7%)	4 (4%)	16 (8.7%)		
≥48 hours	73 (25.5%)	27 (26.7%)	46 (24.9%)		
Duration of symptoms					
<24 hours	193 (67.5%)	70 (69.3%)	123 (66.5%)	χ²(1)=0.24^b^	0.627
≥24 hours	93 (32.5%)	31 (30.7%)	62 (33.5%)		
Door-to-detorsion time (mins)	147 (97, 193)	165 (117, 208)	139 (86, 177)	Z=2.68^a^	0.008
Degree of torsion					
0°	36 (15.3%)	10 (11.6%)	26 (17.3%)	Z=1.31^c^	0.191
180°	32 (13.6%)	10 (11.6%)	22 (14.7%)		
300-450°	99 (41.9%)	39 (45.4%)	60 (40%)		
>540°	69 (29.2%)	27 (31.4%)	42 (28%)		
Side					
Left	135 (47.2%)	48 (47.5%)	87 (47%)	χ²(1)=0.007^b^	0.936
Right	151 (52.8%)	53 (52.5%)	98 (53%)		
Type of surgery					
Orchiopexy	195 (68.2%)	64 (63.4%)	131 (70.8%)	χ²(1)=1.67^b^	0.196
Orchiectomy	91 (31.8%)	37 (36.6%)	54 (29.2%)		

The median duration of symptoms (nine hours) was the same between these two groups. Of the 101 patients before COVID-19, 37 (36.6%) underwent an orchiectomy compared to 54 (29.2%) during or after the peak of COVID-19 (p=0.196). The median DTD was significantly different between pre- and during/post-COVID-19 periods, as indicated by the Wilcoxon rank-sum test (p=0.008). The median DTD was 139 minutes during and after the peak of COVID-19 compared to 165 minutes prior to COVID-19. The features of patients with TT by individual year between 2015 and 2024 are depicted in Table 3. 

**Table 3:** Features of patients with testicular torsion by individual year (January 1, 2015-December 31, 2024) Data presented as median (interquartile range) or n (%). ^a^H=Kruskal-Wallis test statistic (df=9); ^b^χ²=Pearson's chi-squared test statistic (df=9); ^c^M²=Cochran-Mantel-Haenszel test statistic (df=1).

	2015	2016	2017	2018	2019	2020	2021	2022	2023	2024	Test statistics	P-value
	N=20	N=22	N=15	N=20	N=20	N=41	N=41	N=29	N=50	N=28		
Age (yr)	14 (13, 17)	14 (13, 15)	14 (12, 16)	14 (12, 15)	14 (13, 16)	14 (12, 15)	15 (14, 16)	14 (13, 15)	15 (12, 16)	15 (14, 16)	H(9)=12.32^a^	0.196
Duration of symptoms (hr)	9 (5, 22)	12 (5, 72)	7 (6, 24)	11 (4, 48)	9 (5, 34)	16 (5, 48)	6 (3, 24)	8 (3, 48)	12 (4, 48)	10 (4, 29)	H(9)=4.72^a^	0.858
Duration of symptoms												
0 to <6 hours	6 (30%)	7 (32%)	3 (20%)	7 (35%)	7 (35%)	14 (34%)	20 (49%)	10 (34%)	16 (32%)	9 (32%)	M²(1)=0.24^c^	0.625
6 to <12 hours	7 (35%)	3 (14%)	7 (47%)	4 (20%)	6 (30%)	4 (10%)	4 (10%)	5 (17%)	9 (18%)	5 (18%)		
12 to <18 hours	1 (5%)	4 (18%)	1 (7%)	1 (5%)	1 (5%)	4 (10%)	6 (15%)	1 (3%)	4 (8%)	4 (14%)		
18 to <24 hours	1 (5%)	2 (9%)	0 (0%)	0 (0%)	1 (5%)	4 (10%)	0 (0%)	1 (3%)	2 (4%)	2 (7%)		
24 to <48 hours	1 (5%)	0 (0%)	1 (7%)	1 (5%)	0 (0%)	2 (5%)	5 (12%)	2 (7%)	5 (10%)	3 (11%)		
≥48 hours	4 (20%)	6 (27%)	3 (20%)	7 (35%)	5 (25%)	13 (32%)	6 (15%)	10 (34%)	14 (28%)	5 (18%)		
Duration of symptoms												
<24 hours	15 (75%)	16 (73%)	11 (73%)	12 (60%)	15 (75%)	26 (63%)	30 (73%)	17 (59%)	31 (62%)	20 (71%)	χ²(9)=4.89^b^	0.844
≥24 hours	5 (25%)	6 (27%)	4 (27%)	8 (40%)	5 (25%)	15 (37%)	11 (27%)	12 (41%)	19 (38%)	8 (29%)		
Door-to-detorsion time (mins)	150 (94, 227)	161 (121, 220)	180 (121, 216)	174 (126, 289)	156 (88, 187)	130 (76, 160)	126 (85, 163)	141 (101, 159)	151 (92, 188)	155 (88, 219)	H(9)=15.45^a^	0.079
Degree of torsion												
0°	3 (18%)	1 (7%)	3 (23%)	3 (16%)	0 (0%)	6 (16%)	2 (7%)	6 (29%)	9 (23%)	3 (12%)	M²(1)=0.56^c^	0.453
180°	3 (18%)	1 (7%)	3 (23%)	1 (5%)	2 (11%)	2 (5%)	3 (10%)	3 (14%)	9 (23%)	5 (20%)		
300-450°	10 (59%)	8 (53%)	5 (38%)	8 (42%)	7 (37%)	20 (53%)	11 (38%)	7 (33%)	13 (33%)	10 (40%)		
>540°	1 (6%)	5 (33%)	2 (15%)	7 (37%)	10 (53%)	10 (26%)	13 (45%)	5 (24%)	9 (23%)	7 (28%)		
Side												
Left	9 (45%)	13 (59%)	7 (47%)	7 (35%)	10 (50%)	16 (39%)	16 (39%)	13 (45%)	28 (56%)	16 (57%)	χ²(9)=7.48^b^	0.588
Right	11 (55%)	9 (41%)	8 (53%)	13 (65%)	10 (50%)	25 (61%)	25 (61%)	16 (55%)	22 (44%)	12 (43%)		
Type of surgery												
Orchiopexy	14 (70%)	12 (55%)	9 (60%)	15 (75%)	13 (65%)	20 (49%)	34 (83%)	22 (76%)	38 (76%)	18 (64%)	χ²(9)=16.52^b^	0.057
Orchiectomy	6 (30%)	10 (45%)	6 (40%)	5 (25%)	7 (35%)	21 (51%)	7 (17%)	7 (24%)	12 (24%)	10 (36%)		

A borderline statistically significant difference in the annual orchiectomy rate was also identified (p=0.057). A univariate logistic regression assessing the association with the type of surgery (orchiectomy vs. orchiopexy) is presented in Table 4. 

**Table 4:** Univariate logistic regression assessing the association with type of surgery (orchiectomy vs. orchiopexy) in patients with testicular torsion Bolded p-values indicate statistical significance (p<0.05).

	Unadjusted OR	95% CI	Wald χ² (df=1)	P-value
COVID-19 periods (post- vs. pre-COVID-19)	0.713	(0.426, 1.192)	1.663	0.197
Age (yr)	0.86	(0.79, 0.93)	13.2	0.0003
Door-to-detorsion time (every 10 minutes)	1.061	(1.027, 1.096)	12.9	0.0003
Symptom duration (from onset to ED) (hr)	1.03	(1.02, 1.04)	39.52	<0.0001
Degree of torsion: 180° vs. 0°	1.148	(0.300, 4.396)	0.041	0.840
Degree of torsion: 300-450° vs. 0°	2.826	(1.003, 7.961)	3.866	0.049
Degree of torsion: >540° vs. 0°	4.769	(1.656, 13.733)	8.379	0.004

After adjustment for age, DTD, symptom duration, and degree of TT, the COVID-19 period was not significantly associated with orchiectomy (p=0.308). Although the adjusted odds of orchiectomy were 47% higher in the post-COVID-19 period compared with the pre-COVID-19 period, this difference did not reach statistical significance. Each 10-minute increase in DTD was independently associated with a 0.6% increase in the odds of orchiectomy (p=0.0007), regardless of the COVID-19 period (Table 5). 

**Table 5:** Multivariable logistic regression assessing the association of the COVID-19 period with type of surgery (orchiectomy vs. orchiopexy) in patients with testicular torsion Bolded p-values indicate statistical significance (p<0.05).

	Adjusted OR	95% CI	Wald χ² (df=1)	P-value
COVID-19 periods (post- vs. pre-COVID-19)	1.47	(0.70, 3.07)	1.04	0.308
Age (yr)	0.84	(0.74, 0.94)	8.75	0.003
Door-to-detorsion time (every 10 minutes)	1.06	(1.01, 1.11)	5.39	0.020
Symptom duration (from onset to ED) (hr)	1.03	(1.01, 1.04)	19.52	<0.0001
Degree of torsion: 180° vs. 0°	1.38	(0.29, 6.59)	0.17	0.684
Degree of torsion: 300-450° vs. 0°	2.51	(0.69, 9.14)	1.95	0.163
Degree of torsion: >540° vs. 0°	5.08	(1.39, 18.55)	6.04	0.014

Multivariate logistic regression: TT comparing pre- and post-COVID-19 groups (two groups)

Table 6 depicts a multivariable logistic regression model including the interaction between symptom duration and degree of TT. We tested for interactions between key clinical variables; a significant interaction was found between symptom duration and degree of TT (p=0.001), though it did not materially alter the COVID-19 period effect estimate.

**Table 6:** Multivariable logistic regression model including interaction between the symptom duration and degree of testicular torsion Bolded p-values indicate statistical significance (p<0.05).

	Adjusted OR	95% CI	Wald χ² (df=1)	P-value
COVID-19 periods (post- vs. pre-COVID-19)	1.536	(0.71, 3.34)	1.17	0.280
Age (yr)	0.822	(0.73, 0.93)	9.43	0.002
Door-to-detorsion time (every 10 minutes)	1.056	(1.00, 1.11)	4.24	0.040
Symptom duration (from onset to ED) (hr)	1.001	(0.99, 1.01)	0.02	0.887
Degree of torsion: 180° vs. 0°	0.709	(0.12, 4.08)	0.15	0.700
Degree of torsion: 300-450° vs. 0°	0.896	(0.23, 3.43)	0.03	0.873
Degree of torsion: >540° vs. 0°	2.247	(0.61, 8.31)	1.47	0.225
Symptom duration (hr)×degree of torsion (180°)	1.029	(0.99, 1.07)	1.88	0.170
Symptom duration (hr)×degree of torsion (300-450°)	1.039	(1.02, 1.06)	10.39	0.001
Symptom duration (hr)×degree of torsion (>540°)	1.035	(1.01, 1.06)	6.96	0.008

## Discussion

Several studies have evaluated the influence of the COVID-19 pandemic on the duration of symptoms of TT and the number of orchiectomies (Table 7) [8-19]. 

**Table 7:** Pediatric testicular torsion before and during the COVID-19 pandemic in the literature *p=0.007; **p=0.001

Study	Years of study (before (A) and during (B) the COVID-19 pandemic)	Number of patients	Duration of symptoms (median, hours)	Percentage of orchiectomies (%)
Nelson et al. [18]	A: 1/1/2018-2/29/2020	77	5.6	17
	B: 3/1/2020-5/31/2020	17	2.4	29
Holzman et al. [16]	A: 1/2019-2/2020	137	17.9	29
	B: 3/2020-7/2020	84	7.5	43
Lee et al. [12]	A: 3/11-10/1, 2018-2019	55	15.5	50.9
	B: 3/11/20-10/1/2020	27	8	33.3
Littman et al. [17]	A: 3/15-5/4/, 2015-2019	57	23.2	44.7
	B: 3/15-5/4, 2020	21	6	25
Pogorelić et al. [10]	A: 1/1/2019-3/10/2020	68	6*	16.2**
	B: 3/11/2020-12/31/2020	51	14	43.1
Shields et al. [14]	A: 3/1-12/31, 2015-2019	79	8	38
	B: 3/1-12/31, 2020	38	16	50
Yu and Zhang [8]	A: Not specified	13	12	46.2
	B: Not specified	12	2 days	41.7
Zambaiti et al. [9]	A: 1/2019-2/2020	89	5.5	21
	B: 3/2020-12/2020	99	6	23
Komarowska et al. [13]	A: 1/1/2019-12/31/2019	24	6.5	21
	B: 1/1/2020-12/31/2020	20	8.5	35
Fujimoto et al. [19]	A: 12/1/2009-2/29/2020	38	6.4	23.7
	B: 3/1/2020-12/31/2022	9	20	33.3
Current study (2026)	A: 1/1/2015-2/29/2020	101	9	36.6
	B: 3/1/2020-12/31/2024	185	9	29.2

The median symptom duration varied between these studies, with some reflecting a shorter duration during COVID-19 [12,16-18], while others reported a longer duration [8-10,13,14,19]. The longer symptom duration during COVID-19 may be attributed to patients and their families not desiring to seek emergency care in the midst of the pandemic for fear of contracting the virus themselves. Similarly, the percentage of orchiectomies between the studies differed among the extant literature, with several reporting a higher percentage during COVID-19 [9,10,13,14,16,18,19] compared to before the pandemic [8,12,17]. Interestingly, the studies with a longer symptom duration during COVID-19 also had a higher percentage of orchiectomies during COVID-19.

With the three periods delineated into pre-, during-, and post-COVID-19 in our current study, we were able to determine the impact of COVID-19 on pediatric TT. The mean monthly case volume was significantly different across these periods, with the average monthly case volume significantly increasing from the pre-COVID-19 period (2.06) to during-COVID-19 (3.69) and post-COVID-19 (2.95) periods (p<0.0001). These findings confirm the significant increase in the number of TT cases during COVID-19 compared to before the pandemic. There was a subsequent tapering in the number of TT cases after the peak of the pandemic, although the number did not decline to that prior to the pandemic. The median DTD was also significantly different across the three COVID-19 periods, with the shortest time occurring during the COVID-19 period (p=0.011). This finding is quite surprising and reveals the competency and efficiency of the pediatric emergency and urological teams in evaluating and treating pediatric patients with TT in the tumultuous times during the height of COVID-19.

Comparing pre- and post-COVID-19 in our current study, the duration of symptoms was both nine hours, and the orchiectomy percentage was higher before COVID-19 compared to during and after the height of COVID-19 (36.6% versus 29.2%) although it was not statistically significant. Despite pandemic disruptions, including stay-at-home mandates and hesitancy to seek medical care for fear of contracting COVID-19, there was no meaningful impact of COVID-19 on TT surgeries. Possible reasons for this finding include that TT remained a high-priority emergency with minimal delays in care as well as the development of compensatory measures where the hospital has maintained effective pathways for time-sensitive surgeries. Providers can be confident and reassured that baseline orchiectomy rates were not worsened by pandemic-related factors.

An additional feature highlighted by a few of these previous studies is the increased number of pediatric patients evaluated during the COVID-19 period [18]. In Nelson et al.'s study, the number of TT case presentations per week increased from 0.7 cases/week in the pre-COVID-19 period to 1.3 cases/week during COVID-19 (p=0.015) [18]. These authors suggested that these higher numbers may have been attributed to diversion or transfer of patients from outside facilities due to the pandemic [18]. In our previous study comparing pediatric TT pre-COVID-19 and during COVID-19, there was a statistically significant number of cases during the pandemic compared to equivalent time periods in the five years prior to COVID-19 (38 versus 15.8; p=0.05) [14]. In our current study, the mean number of patients who underwent surgery for TT per month before COVID-19 (2.06) significantly increased during and after COVID-19 (3.43) (p<0.0001). Contrarily, two extant studies did not report a greater number of pediatric TT cases during the pandemic [12,13]. Lee et al. did not have a higher incidence of pediatric TT during the pandemic compared to before (3.86 cases/month and 3.93 cases/month, respectively) [12]. Komarowska et al. reported a lower number of cases during the pandemic with 20 cases compared to 24 cases prior to COVID-19 [13].

Three systematic reviews and meta-analyses (SR/MA) highlighted pediatric patients with TT before and during the COVID-19 pandemic [20-22]. In Pogorelić and colleagues' SR/MS of 711 patients (473 during COVID-19) from six studies, there were no significant differences in the average duration of symptoms, proportion of children with delayed presentation, and orchiectomy rate before and during the COVID-19 pandemic [21]. These authors acknowledge the moderate level of bias among the previously published studies that may limit the accurate impact of COVID-19 on TT [21]. In Kim and Ahn's SR/MS of 736 patients from seven studies, the symptom duration and orchiectomy rates revealed no significant differences prior to and during the COVID-19 pandemic which conflicted with the authors' hypothesis that the symptom duration would be higher due to the delays in healthcare created by the pandemic [22]. In Ye and colleagues' more expansive SR/MA of 899 patients from eight studies, a statistically significant increased hospitalization rate for patients with TT was observed during the pandemic compared to before its initiation [20]. Although there was a significant increase in symptom duration during COVID-19, orchiectomy rates did not escalate. These authors attributed the stability of orchiectomy numbers during COVID-19 to the efficiency of pediatric emergency services despite pandemic-related closures and delays in transporting patients to medical facilities [20].

Another important finding of the current study is the statistically lower median DTD of 139 minutes during and after the peak of COVID-19 compared to 165 hours before the pandemic. As this time reflects the timeliness between ED presentation and surgery, it is encouraging that the DTD was shorter during COVID-19 despite the chaotic environment of the ED and entire hospital due to patient surges in patients as well as widespread staffing and supply shortages. Our findings differed from other studies that showed a similar DTD before and during COVID-19 [9,12,13,16], despite rapid COVID-19 testing upon ED arrival which may have delayed the DTD [16]. Some patients in our study had a zero-degree TT at the time of surgical exploration. Zero-degree TT means that the pediatric urologist did not see evidence of TT during the surgery (orchiectomy and orchiopexy). The TT may have resolved prior to surgery which may have been due to manual detorsion within the ED. Our study included patients who had evidence of TT at clinical presentation and confirmed by US. Obvious TT may not have been observed during the surgery. Notably, we also determined that the effect of symptom duration on orchiectomy risk varied by the degree of TT, with more severe torsion showing steeper risk per hour (interaction p=0.001). This suggests that torsion severity modifies the time sensitivity of intervention. However, for clinical guidance, our main effects model provides a conservative estimate applicable across torsion severities.

**Figure 1:**
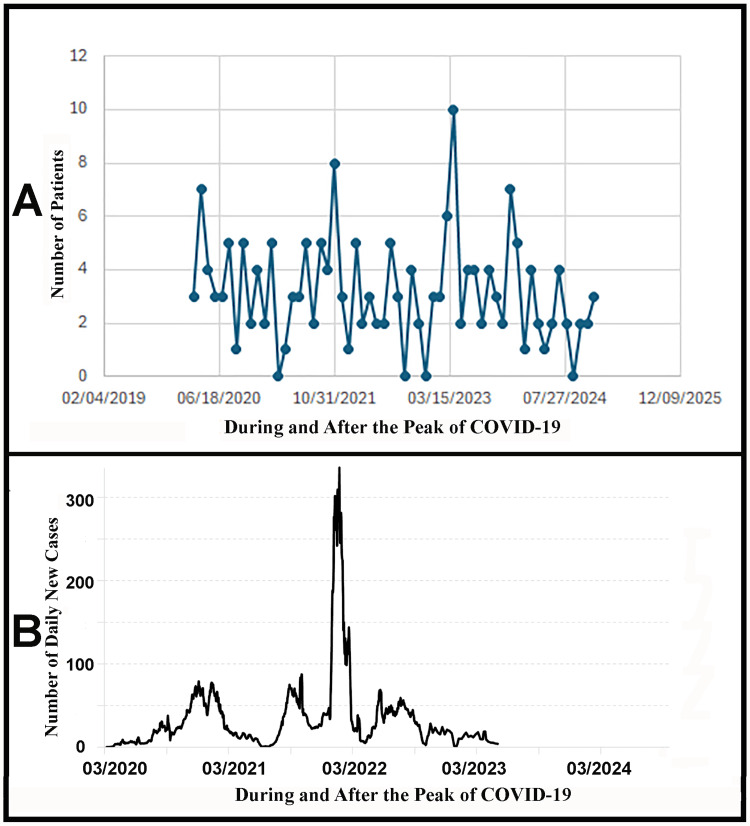
Number of surgeries performed for testicular torsion at our institution compared to the timeline of COVID-19 cases in Jefferson County, Kentucky (A) Number of surgeries performed for testicular torsion at our institution between March 2020 and December 2024. (B) Timeline of COVID-19 cases in Jefferson County, Kentucky, where our institution is located between March 2020 and May 2023. Source for the data in (B) is the following: [22].

Figure 1A highlights the number of surgeries performed for TT at our institution between March 2020 and December 2024. The month with the largest number of TT surgeries was April 2023, following the surge of COVID-19 cases documented in our state. However, the second highest month of TT surgeries was November 2022, two months prior to the greatest number of Kentucky COVID-19 cases reported in January 2023. Figure 1B depicts the timeline of COVID-19 cases in Jefferson County, Kentucky, where our institution is located between March 2020 and May 2023. In Jefferson County, Kentucky, the peak day for new COVID-19 cases was January 22, 2022, with 335.6 daily new cases per 100,000 [23,24]. This height of COVID-19 was attributed to the Omicron variant surge.

While Nelson and colleagues suggested that the increased number of pediatric TT cases may be due to diversion or transfer of patients secondary to pandemic-related factors, we posit that the increased number of pediatric TT cases during the pandemic may be a result of the SARS-CoV-2 virus itself and its preference for invading the testis. Several mechanisms have been proposed for the impact of SARS-CoV-2 on testicular function [25]. COVID-19 may enter the host cell by binding to the ACE2 receptor which is highly expressed in the testis, specifically, the Sertoli and Leydig cells and spermatogonia [26-28]. In Costa and colleagues' study of testicular abnormalities in deceased COVID-19 patients, infiltrative infected monocytes migrate into the testicular parenchyma [25]. SARS-CoV-2 preserves its replicative and infective abilities after a patient's infection, leading these authors to suggest that the testes may represent a viral sanctuary. Infected testes demonstrated thickening of the tunica propria, germ cell apoptosis, Sertoli cell barrier loss, angiogenesis, hemorrhage, Leydig cell inhibition, fibrosis, and inflammation [25]. High angiotensin II levels and activation of mast cells and macrophages may spur the development of testicular pathology. The sperm concentration, total sperm count, and total motility are also significantly lower in patients with COVID-19 infection compared to healthy controls [29]. SARS-CoV-2 has been reported to induce a cytokine storm as the driving factor in testicular injury [30]. COVID-19 also may disrupt the blood-testis barrier through the induction of inflammatory cytokines and impair junctional proteins [31]. The findings of testicular injury and altered serum fertility markers in patients with COVID-19 may signal adverse consequences for reproductive health which is a concern in the pediatric population [25,30,31].

Strengths and limitations

The strength of the current study is the large number of children and adolescents with TT over a 10-year duration. Our current study, encompassing patients before, during, and after the peak of COVID-19, provides a valuable insight into the impact of COVID-19 on pediatric TT over the entire spectrum. The extant literature on the comparison of pediatric TT pre-COVID-19 and during the pandemic only extended until December 31, 2020. The importance of the present study is that it depicts pediatric TT in the aftermath of COVID-19 in 2023 and 2024, after the pandemic was no longer considered an international public health emergency. Our study also showed a significant difference in the median DTD across the three time periods (before, during, and after the COVID-19 pandemic). The shortest DTD occurred during the COVID-19 period. The limitations of this study include its retrospective nature with its associated inter-observer bias among the treating physicians of pediatric patients with TT. There was also a single-center bias which limits the generalizability of the results. Additionally, the SARS-CoV-2 hypothesis was not virally tested, and there was a lack of long-term follow-up of the patients who underwent surgery for TT.

## Conclusions

The COVID-19 pandemic was an international public health emergency which significantly affected healthcare delivery. In this study, we wanted to determine the number of pediatric TT cases and types of surgery performed for this urgent condition following the height of the COVID-19 pandemic. With our cohort categorized into pre-, during-, and post-COVID-19 periods, the number of patients with TT significantly increased during the height of the COVID-19 pandemic, with a subsequent decline. The type of surgery and symptom duration were not statistically different between the three time periods. Our study reflects that pediatric patients and their families sought timely medical intervention for TT despite the multiple public health, social, and psychological challenges facing humanity generated by the COVID-19 pandemic. 
